# Underlying Susceptibility to Eating Disorders and Drug Abuse: Genetic and Pharmacological Aspects of Dopamine D4 Receptors

**DOI:** 10.3390/nu12082288

**Published:** 2020-07-30

**Authors:** Luca Botticelli, Emanuela Micioni Di Bonaventura, Fabio Del Bello, Gianfabio Giorgioni, Alessandro Piergentili, Adele Romano, Wilma Quaglia, Carlo Cifani, Maria Vittoria Micioni Di Bonaventura

**Affiliations:** 1 School of Pharmacy, Pharmacology Unit, University of Camerino, via Madonna delle Carceri, 9, 62032 Camerino, Italy; luca.botticelli@unicam.it (L.B.); emanuela.micioni@unicam.it (E.M.D.B.); mariavittoria.micioni@unicam.it (M.V.M.D.B.); 2 School of Pharmacy, Medicinal Chemistry Unit, University of Camerino, via S. Agostino, 1, 62032 Camerino, Italy; fabio.delbello@unicam.it (F.D.B.); gianfabio.giorgioni@unicam.it (G.G.); alessandro.piergentili@unicam.it (A.P.); wilma.quaglia@unicam.it (W.Q.); 3 Department of Physiology and Pharmacology “V. Erspamer”, Sapienza University of Rome, P.le Aldo Moro 5, 00185 Rome, Italy; adele.romano@uniroma1.it

**Keywords:** dopamine D4 receptor (DRD4), drug addiction, food addiction, eating behavior, 7-repeat allele, obesity, prefrontal cortex, reward, DRD4 agonist, DRD4 antagonist

## Abstract

The dopamine D4 receptor (DRD4) has a predominant expression in the prefrontal cortex (PFC), brain area strictly involved in the modulation of reward processes related to both food and drug consumption. Additionally, the human DRD4 gene is characterized by a variable number of tandem repeats (VNTR) in the exon 3 and, among the polymorphic variants, the 7-repeat (7R) allele appears as a contributing factor in the neurobiological mechanisms underlying drug abuse, aberrant eating behaviors and related comorbidities. The 7R variant encodes for a receptor with a blunted intracellular response to dopamine, and carriers of this polymorphism might be more tempted to enhance dopamine levels in the brain, through the overconsumption of drugs of abuse or palatable food, considering their reinforcing properties. Moreover, the presence of this polymorphism seems to increase the susceptibility of individuals to engage maladaptive eating patterns in response to negative environmental stimuli. This review is focused on the role of DRD4 and DRD4 genetic polymorphism in these neuropsychiatric disorders in both clinical and preclinical studies. However, further research is needed to better clarify the complex DRD4 role, by using validated preclinical models and novel compounds more selective for DRD4.

## 1. Introduction

Dopamine (DA) is a catecholamine neurotransmitter expressed both in the mammalian central nervous system (CNS) and in a variety of peripheral tissues. Within the brain, four principal dopaminergic networks have been identified: (I) the nigrostriatal pathway that includes neurons projecting from the substantia nigra pars compacta to the caudate putamen in the dorsal striatum, whose degeneration is responsible for Parkinson’s disease [1,2]; (II) the mesolimbic pathway, including projections from the ventral tegmental area (VTA) to the nucleus accumbens (NAc) and olfactory tubercles, particularly involved in reward processes [3]; (III) the mesocortical network, that originates from neurons in the tegmentum and projects to the frontal cortex, mainly in the medial prefrontal regions, implicated in cognitive functions and motivation [4]; (IV) the tuberoinfundibular pathway that arises in the arcuate nucleus and ends in the median eminence of the hypothalamus, implicated in the neuroendocrine regulation and in the prolactin release [5,6,7,8]. In peripheral organs, DA is involved in several physiological functions including vision, olfaction, cardiovascular, hormonal and sympathetic regulation [5,9]. DA acts by binding five distinct receptors that are members of the G-protein coupled receptor superfamily. These proteins are characterized by seven hydrophobic transmembrane regions and a crucial third intracytoplasmic loop that interacts with different types of G-proteins and multiple effector molecules [5,7,9]. According to the biochemical and pharmacological features, these receptors are divided into the D1-like family, which includes D1 and D5 receptor subtypes (DRD1 and DRD5), and the D2-like family, comprising D2, D3 and D4 receptor subtypes (DRD2, DRD3 and DRD4). D1-like receptors activate the enzyme adenylyl cyclase, through a Gs-type protein, increasing levels of the second messenger cyclic adenosine monophosphate (cAMP), while D2-like receptors, coupling to a Gi or Go protein, inhibit the adenylyl cyclase, reducing cAMP concentrations [5,8,10,11]. In addition to the ability to modulate cAMP levels through adenylyl cyclase, D1-like and D2-like receptors are also able to activate other transductional signaling pathways. In particular, the stimulation of D1-like receptors results in the mobilization of intracellular calcium stores via the activation of phospholipase C, while the stimulation of D2-like receptors increases potassium and suppresses calcium currents, leading to cell hyperpolarization [8,9,10,11].

Within the D1-like class, DRD1 has the highest levels of expression in the brain: specifically, DRD1 mRNA has been found in neurons of the caudate putamen, olfactory tubercule, NAc, amygdala, substantia nigra and frontal cortex; low levels were also detected in hippocampus and thalamic areas [5,7,11]. In contrast, DRD5 are less expressed compared to the DRD1 subtype and are predominantly found within the hippocampus, parafascicular nucleus of the thalamus, cerebral cortex and dentate gyrus [5,9,12,13,14].

With regard to D2-like family, the main localization of the DRD2 is in the caudate putamen, olfactory tubercle and NAc, and the expression was also detected in the substantia nigra pars compacta and in the VTA [8,10]. The distribution of DRD3 is limited to the limbic area, olfactory tubercle and island of Calleja and, at lower levels, in the substantia nigra and VTA [7,8,9]. Finally, the expression of the DRD4 has been found predominantly in the frontal cortex, hippocampus, amygdala, hypothalamus and mesencephalon, while lower levels were detected in the basal ganglia [5,8,9,15].

Despite the limited expression in the brain compared to the other receptor subtypes, the DRD4 is particularly interesting because of the highly polymorphic nature of the human gene encoding for this protein [16,17] and because several human studies revealed DRD4 possible involvement in neuropsychiatric diseases, including attention deficit hyperactivity disorder (ADHD) [18,19], personality traits of novelty seeking [20,21], schizophrenia [22,23], psychostimulants addiction [24], mood disorders [25], eating disorders (ED) [26,27,28] and obesity [29,30]. In this review, after a brief description of the DRD4, we will revise the current literature linking this receptor and DRD4 gene polymorphism to the development of drug addiction and aberrant ED, including binge eating behavior and obesity, describing the possible role played by the DRD4 in neural mechanisms implicated in these neuropsychiatric disorders.

### The Dopamine D4 Receptor

The DRD4 is encoded by the DRD4 gene, localized on chromosome 11p15.5. This gene contains four exons and is highly polymorphic in the coding sequence. The most extensive polymorphism is present in the Exon3, a region that codes for the putative third intracytoplasmic loop of the receptor [16,17,31]. This polymorphism is characterized by a variable number of tandem repeat (VNTR), in which a 48-base-pair sequence exists as 2- to 11-fold repeat [15,16,17,31]. Allele frequencies of polymorphic variants of the DRD4 gene differ among several human populations, with the 4-repeat (4R) being the most common (64%), followed by the 7-repeat (7R) (21%) and the 2-repeat (2R) alleles (8%) [15,31]. Interestingly, it has been demonstrated that VNTR polymorphism influences DRD4 coupling to the adenylyl cyclase; in fact, DA is twice as potent in the blockade of forskolin-induced cAMP accumulation on 2R and 4R than on 7R receptor variant, suggesting that the 7R allele encodes for a receptor with a reduced affinity for DA [16,32].

Although DA is the most potent endogenous ligand able to activate the DRD4, also noradrenaline and adrenaline, at submicromolar concentrations, activate DRD4, while histamine and serotonin do not show significant affinity for this receptor [33,34]. The activation of DRD4 not only inhibits cAMP production but also opens the Kir3 potassium channel, induces arachidonic acid release, activates extracellular signal-regulated kinase ERK1 and 2 and reduces the level of functional GABAa receptors [15].

The distribution pattern of DRD4 in the brain principally involves the cerebral cortex, amygdala, hippocampus, hypothalamus, and pituitary gland and, even though at lower levels, the basal ganglia, as assessed through Northern Blot and RT-PCR [35,36,37], in situ hybridization [38,39], and immunohistochemistry studies [40,41,42,43,44]. Moreover, high concentrations of DRD4 mRNA were detected also in the human retina [35]. The expression profile of the DRD4 is not limited to the CNS, considering that it was also found in the cardiac atrium [45], lymphocytes [46] and kidney [47].

The neuroanatomical profile of the DRD4 is different from the other DA receptor subtypes, strongly expressed in the striatum [48], because the predominant localization is in the prefrontal cortex (PFC) [40,42,43]. The PFC is a brain region associated with executive functions, such as working memory, novelty seeking and emotional processes [49,50]; specifically, the DRD4s are positioned on inhibitory GABAergic interneurons and on excitatory glutamatergic pyramidal neurons, including the striatal projections [51,52,53].

This localization is an important evidence of DRD4 ability to modulate GABAergic neurotransmission [43]. Additionally, a recent preclinical study reported that DRD4s, situated postsynaptically on pyramidal neurons, are able to modulate glutamatergic corticostriatal neurotransmission [54]. The significant role of the DRD4 as a modulator of the neural network activity was confirmed by studies using mice lacking the DRD4, which demonstrated an increased cortical hyperexcitability in the frontal cortex [55] and hypervigilant state and abnormal behavioral responses to environmental stimuli compared to wild-type mice (WT) [56]. These findings suggest that DRD4 has a critical role in the regulation of neurotransmission at the level of frontocortical areas of the brain and the dysregulation of dopamine DRD4-mediated signaling might be responsible of several pathological neuropsychiatric conditions.

## 2. DRD4 and Drug Addiction

Addiction is defined as a chronically relapsing disorder characterized by three main stages: (1) compulsive drug seeking and intake, (2) loss of control in limiting drug intake, (3) emergence of a negative emotional state (e.g., anxiety, dysphoria, irritability) when access to the drug is precluded [3].

The mesolimbic DA pathway is the principal neural network involved in reward processes and includes projections from the VTA to the NAc, but other pathways are implicated in drug addiction, the mesocortical (projections from VTA to the frontal cortex) and the mesostriatal (projections from the substantia nigra to the dorsal striatum) [57]. The rewarding effects of drugs of abuse are associated with the ability to release DA in the NAc, located in the ventral striatum [58], and the amount of released DA is correlated to the subjective perception of the drug as rewarding [59].

Due to the involvement of DA in motivational processes, the increased DA signaling associated with drugs and drug-related cues is able to enhance the motivation to obtain the reward [60]. In fact, according to “incentive salience hypothesis” proposed by Berridge and Robinson, DA assigns “incentive salience” to objects and behavioral acts and converts the neural representation of a stimulus into an attractive object that animals will try to obtain [61]. Thus, the reinforcing effect of drugs of abuse is due not only to the pleasurable property but mainly to the fact that, increasing DA transmission, they are considered as salient stimuli that will stimulate consumption of more drug, regardless of whether it is pleasurable or not [59].

Repeated exposure to substances of abuse results in neuroadaptations of the brain reward system and, in particular, it is observed a significant reduction of DRD2 and of DA released by DA cells [62]. Changes of DRD2 levels in addictive individuals in the striatum also contribute to reduced activity of prefrontal regions, including the anterior cingulate cortex, the orbitofrontal cortex and the dorsolateral PFC [63], where also DRD4 is widely expressed [40,41,42], and this neuroadaptation of frontocortical areas may be responsible of compulsive drug-seeking behaviors [64]. The PFC is a heterogenous area of the brain that is implicated in reward processes, due to its known role in value-based decision making and in inhibitory control [65]. The three regions of the PFC principally involved in the reward system are the dorsal anterior cingulate cortex, the ventromedial PFC and the orbitofrontal cortex [66]. It has been observed that the PFC sends glutamatergic projections to the VTA and thus exerts an excitatory control, by inducing cell firing of dopaminergic neurons, an event considered crucial for the central effects of drugs of abuse [67]. As mentioned above, DRD4 modulates glutamatergic corticostriatal neurotransmission [54], and since the VTA projects to the basal ganglia, it is believed that glutamatergic projections from the PFC could be responsible for the onset of incentive salience [68], process in which the DRD4 could be deeply involved. In fact, the exposure to drug-related cues induces activation of glutamatergic projections from the PFC, hippocampus and amygdala to striatal projections, leading to an increased DA release in the NAc and dorsal striatum [69]. Moreover, the PFC, essential in processing and filtering emotionally relevant sensory information, modulates input from the VTA and the amygdala, and this neuronal pathway is involved in encoding learned associations among sensory stimuli and emotionally salient events [51,70,71]. Disruption of this pathway, in which DRD4 seems to play a critical role [72,73], can lead to psychiatric disorders, including drug dependence, food addiction and ED.

Due to the predominant localization of the DRD4 in the PFC [74] and the involvement of the DRD4 gene polymorphism with personality traits of novelty seeking, impulsivity and risk-taking behaviors [20,21,75], the DRD4 has been extensively studied in the context of drug addiction. In the next paragraphs we will analyze genetic studies linking the DRD4 gene to substance abuse and preclinical studies that investigated the role of this receptor in drug dependence.

### 2.1. Polymorphism of DRD4 Gene and Drug Addiction

The highly polymorphic nature of the DRD4 gene seems to be a potential genetic susceptibility factor to the development of substance abuse and is correlated with addiction and addiction associated-phenotypes [76,77,78].

Hutchinson et al. found that carriers of the 7R allele of the DRD4 gene or longer (DRD4 L) had more “urge to drink” and an increased craving after alcohol consumption than carriers of short allele (DRD4 S) [79]. DRD4 L individuals also reported less subjective stimulation and euphoria following alcohol administration, suggesting that they could be more motivated to engage activities that induce DA release [79]. Polymorphic variants of the DRD4 gene appear also to influence pharmacological efficacy in the treatment of Alcohol Use Disorder: it was demonstrated that olanzapine, an atypical antipsychotic, which is a DRD4 and DRD2 antagonist with also high affinity for serotonergic, muscarinic, histamine, and α-1-adrenergic receptors, reduced craving elicited by alcohol cues only in DRD4 L carriers, but not in DRD4 S carriers [80,81]. Indeed, Naltrexone, an opiate antagonist used in the treatment of alcohol dependence [82], decreased heavy drinking and craving during non-drinking times only in DRD4 L carriers [83,84]. Moreover, Fibey et al. found a greater activation of the striatum, orbitofrontal cortex and anterior cingulate in carriers of the DRD4 L before a priming dose of alcohol but not after the administration [77,85]. Other works demonstrated an association between 7R allele and excessive alcohol intake, proposing that this relationship could be mediated by personality traits of novelty seeking [86,87]. A similar result was obtained by analyzing smoking behaviors and novelty seeking in adolescents with or without the 7R allele [88]. In accordance with previous observations, in participants that performed an alcohol/money choice task after cue exposure, the presence of the 7R variant was associated with a greater valuation of alcohol [89]. However, a non-significant influence between DRD4 gene polymorphism and reactivity to alcohol-related cues has been observed by van den Windelberg et al. [90].

The 7R variant of the gene appears to affect also smoking behaviors and nicotine dependence, as demonstrated by Shields et al. study, in which African American individuals, but not Caucasian people, with this polymorphism, showed a high risk of smoking [91]. Carriers of this allele reported greater functional magnetic resonance imaging (fMRI) signal in brain regions like the right superior frontal gyrus of the PFC and right insula compared to non-carriers in response to smoking cues [92]. In line with these results, a previous study demonstrated that DRD4 VNTR polymorphism influences reactivity to smoking cues, and in particular DRD4 L individuals reported a significant increase in craving, attention and arousal during the exposure to smoking cues compared to DRD4 S individuals [93]. Smokers with the 7R allele consumed more cigarettes per day, reported both less reduction of craving and lower DA release in the ventral caudate and NAc after smoking [94]. Several works also reported that the presence of at least one copy of the DRD4 L was linked to a reduced likelihood to smoking cessation [95,96] and to an increased reactivity to smoking related cues in ex-smokers, suggesting an influence of DRD4 VNTR in the abstinence from nicotine [97].

Additionally, the DRD4 L was correlated with an increased craving after heroin-related stimuli in Chinese heroin addicted population [98] and in Israeli male opioid-dependent subjects, compared to control group with no history of substance abuse [99], supporting the hypothesis that the 7R allele represents a potential susceptibility factor for the development of opioid dependence [100].

Finally, methamphetamine abusers revealed a significantly higher prevalence of the 7R allele than control subjects [101]; however, a different result was reported in Chinese male population of methamphetamine dependents, in which the presence of the DRD4 gene polymorphic variant did not seem to be relevant [102].

All together, these studies highlight the important role played by the DRD4 gene in the development of drug addiction. The presence of the 7R allele variant might be associated with a blunted DA signaling [32], leading to an increased susceptibility to addictive behaviors.

This speculation can be explained by the “reward deficiency syndrome”, hypothesis in which individuals with a genetic predisposition to a deficient DA signaling in the reward system may be more tempted to enhance DA levels in the brain by the repetitive administration of drugs of abuse [103]. Genetic association studies are particularly important to understand the role played by the DRD4 in drug addiction, together with preclinical studies, using genetically modified mice and evaluating DRD4 selective compounds, discussed in the next section.

### 2.2. Preclinical Studies

Experiments with genetically modified mice, in particular DRD4 KO mice, support a possible role of the DRD4 in drug abuse. In 1997, Rubinstein et al. found that mutant mice completely lacking functional DRD4 showed a less spontaneous locomotor activity but were supersensitive to the locomotor stimulating effects of ethanol, cocaine and methamphetamine [104]. Locomotor activity is correlated with the addictive properties of psychostimulants [105], and the following studies reported similar results, confirming that DRD4 deficient mice exhibit a heightened locomotor sensitivity in response to psychostimulants [106,107,108]. Conversely, Thanos et al., measuring locomotor activity in conditioned place preference (CPP) chambers, found in DRD4 KO mice an attenuated response to amphetamine at the dose of 1 mg/kg, but the same mice exhibited a greater response at the dose of 3 mg/kg compared to WT and heterozygotes, while no difference was observed in response to methylphenidate and cocaine among all genotypes [109]. In the same experiment, the effect of these psychostimulants was also evaluated on CPP. The CPP test is a well-validated protocol for the evaluation of the rewarding and adversive effects of drugs [110]. DRD4 KO mice treated with methylphenidate and amphetamine showed significant CPP, meanwhile treatment with cocaine had no significant effect compared to the other genotypes [109]. This evidence is in line with a recent study, in which the DRD4 seems not to play a significant role in acquiring CPP for cocaine, but it could affect extinction and reinstatement for CPP [111].

DRD4 appears to further affect novelty seeking, a behavior strongly associated with the increased risk of drug addiction [112,113,114], even though controversial results were reported. In Dulawa et al. work, DRD4 KO mice reduced the behavioral responses to novel stimuli, using three tests: open field, emergence and novel object test [115]. Indeed, the stimulation of the DRD4 with the DRD4 partial agonist RO-10-5824 was able to increase novel object exploration in mice [116].

On the contrary, in another study, DRD4 KO and WT mice did not significantly differ in novel object exploration and impulsivity [117]. Subsequently, Thanos et al. reported that a deficient DRD4 signaling was associated with increased novelty seeking behavior in mice, but this effect was sex dependent and mainly observed in male mice [118]. In the same study, DRD4 depleted mice were less aggressive than control WT mice in a social interaction test, but became more aggressive after alcohol withdrawal [118]. Similarly, in humans the 7R allele of the DRD4 gene is associated with a higher craving following alcohol consumption [79] and craving is positively correlated with aggressive behaviors [119].

Mutant mice lacking DRD4 offer an interesting and important tool to understand the role of the DRD4 in the neurobiology of drug addiction, but results should be carefully interpreted, considering the compensatory changes observed in the brain of DRD4 deficient mice [120].

#### Comparison Between DRD4 Antagonists and Agonists in Drug Addiction

The possible role of the DRD4 in drug addiction led researchers to evaluate the effects of DRD4 selective compounds on drug-taking behaviors in animal studies.

The highly selective DRD4 antagonist L-745,870 (>2000-fold and >5000-fold selectivities over, respectively, DRD2 and DRD3, and >20,000-fold selectivity over DRD1 and DRD5 and a moderate affinity for sigma, 5-HT2 and α-adrenergic receptors [121,122]) did not show efficacy in the reduction of the discriminative stimulus of cocaine in rats [123,124]. However, positive results were obtained in other studies, in which the blockade of DRD4 with the same antagonist attenuated methamphetamine-induced discriminative effect in rats [125,126] and morphine-induced withdrawal symptoms precipitated by naloxone [127]. Additionally, a recent study by Kim et al. reported that L-745,870 decreased alcohol self-administration and stress-induced reinstatement, without influencing cue-induced reinstatement [128].

The same DRD4 antagonist was able to attenuate reinstatement of both nicotine- and cue-induced nicotine seeking behavior [129] even though ineffective in decreasing nicotine self-administration, suggesting the possible efficacy of DRD4 antagonists in the prevention of tobacco smoking relapse.

The inability of L-745,870 to reduce nicotine self-administration can be assigned to the poor expression of DRD4 in the NAc [31,42], proposing a limited effect in the modulation of the reinforcing properties of drugs of abuse. On the contrary, the significant effect in the reduction of reinstatement for nicotine can be explained by the DRD4 predominant localization in brain regions like the insular cortex or the frontal cortex. The activation of these brain areas has been critically correlated with the exposure to nicotine-related cues and reinstatement of nicotine-seeking behaviors [130,131].

Moreover, intraaccumbal admninistration of L-750,667, another DRD4 antagonist (>2000-fold selectivity over DRD2 and DRD3 and little affinity for DRD1, DRD5, sigma, 5-HT1A and 5-HT2 receptors [132]), failed to attenuate reinstatement of cocaine-seeking behaviors in rats [133], confirming the role of the DRD4 in relapse to drugs of abuse that is principally mediated by cortical regions of the brain and marginally in accumbal regions. The DRD4 also influences behavioral sensitization induced by psychostimulants. In fact, the blockade of the DRD4 with the selective antagonist PNU-101387G, with high affinity for DRD4 and relatively low for the other monoamine receptors, prevented amphetamine induced-behavioral sensitization [134].

Taking into account these results, DRD4 antagonists can be considered in the treatment of drug addiction, since they prolong abstinence and do not increase drug-self administration [24]. Thus, the blockade of DRD4 may reduce the risk of abuse liability in humans by influencing motivational processes associated with drug-intake [24,128].

It cannot be excluded that pharmacological activation of the DRD4 might also be a useful approach to treat drug addiction, considering that the DRD4 agonist PD-168,077 (>400-fold selectivity over the DRD2 and >300-fold selectivity versus DRD3 but affinity also for α-adrenergic receptors as well as 5-HT1A and 5-HT1B receptors [135,136]) was able to decrease morphine-induced hyperlocomotion, reward and withdrawal syndrome, without interfering with the analgesic properties [137]. This activity seems to be caused by a functional interaction between DRD4 and μ-opioid receptor [138] in the striosomal compartments of the caudate putamen and substantia nigra pars reticulata, regions in which these receptors are co-expressed [44,139]. In fact, the stimulation of DRD4 with a selective agonist is able to reduce the activation of the nigrostriatal pathway induced by morphine, to restore DA tone [137] and to prevent morphine-induced expression of several transcription factors as well as long-term μ-opioid receptor sensitization in the caudate putamen [140,141,142,143].

Moreover, DRD4 stimulation does not exhibit reinforcing properties, conversely to psychostimulant drugs [24]. In fact, the DRD4 agonist ABT-724, tested in rhesus monkeys trained to self-administer cocaine, was not able to maintain the rates of self-administration behavior [144]. However, the inefficacy of this compound was probably due to the profile of partial agonist, while a full agonist could have been more effective [144]. Accordingly, A-412997, another DRD4 agonist (>300-fold selective over other DA receptors, and 125-fold over 5-HT1A receptors [136]), did not induce CPP in rats [145] in contrast to amphetamine, not resulting in reward related behaviors. These findings confirmed that the stimulation of the DRD4 did not serve a reinforcing function, suggesting that the DRD4 could be an innovative target for the development of new therapeutic strategies for drug addiction and other addictive behaviors. All compounds were summarized in Table 1.

## 3. DRD4 and Feeding Behavior

The DRD4 gene polymorphism seems to affect the cue-elicited craving not only for drugs of abuse but also for natural stimuli, such as food [146]. Interestingly, an overlap between the neural mechanisms implicated in the appetitive motivation for drugs and those involved in appetitive motivation for food was reported, supporting the “food addiction” hypothesis [146,147,148,149]. Accordingly, both drugs of abuse and food consumption (especially addictive food) activate the same brain pathway involved in reward and motivation [150], where the dopaminergic signaling is the principal neurotransmission system; the increase of DA levels in those brain areas could reinforce the effects of addiction [151].

As mentioned above, the DRD4 is the only D2-like subtype strongly expressed in PFC [40,42,43], brain region that modulates drug craving, but also the rewarding properties of foods [152]. In fact, the PFC has anatomical connections with feeding-related areas, including the lateral hypothalamus, NAc shell and limbic reward sites [153,154]. The PFC circuitry is linked with cognitive aspects of searching for food [155], self-control, salience attribution and awareness [156], reward-based decision-making [66], and a dysregulation of such circuit might lead to the development of obesity [157,158,159,160]. In keeping with this latter observation, different imaging studies suggested the PFC activation in response to food-related cues [161,162,163]; such activation is also implicated in yohimbine-induced reinstatement of food seeking in rodents [164,165].

Furthermore, the PFC is involved in stress response [166,167] and interacts with the hypothalamic-pituitary adrenal (HPA) axis [168]. Specifically, Armbruster et al. examined the impact of DRD4 polymorphism on HPA activity, showing a significant smaller cortisol response to social stress in healthy adult carriers of the 7R allele [169]. Cortisol reactivity and the stress neurohormone corticotropin-releasing factor (CRF) effects, via HPA axis and extrahypothalamic brain site, may be a marker for vulnerability to stress, inducing binge eating and subsequent ED. Remarkably, the episode of binge eating is associated with high cortisol levels [170,171,172,173,174,175] that predict sweet food overeating [176], and CRF receptors were demonstrated to play a pivotal role in stress induced binge eating in several studies [177,178,179,180,181,182,183,184,185].

These considerations on DRD4 distribution and the role in drug dependence, previously debated, provide evidence on the potential implication of DRD4 signaling in ED and obesity.

Over the last few years, considerable progress has been made to understand the neurobiological mechanisms that underlie ED, converging that genetic factors and gene/environment interactions could contribute to the potential development of these disorders [186,187,188,189]. One of the possible candidates that may contribute to the dysregulation of feeding patterns in ED and risk of weight gain, leading to obesity, is the polymorphism of DRD4 gene. In this context, several studies identified the genetic variability of DRD4 as a factor in binge eating behavior, bulimia nervosa (BN) and anorexia nervosa (AN) as well as overweight [26,27,28,190]. Binge eating is a typical symptom in ED, in particular BN, binge/purging subtype of AN and binge eating disorders (BED), characterized by an excessively compulsive intake of palatable food in a discreet period of time [191]. Differently from AN and BN, individuals with BED do not engage in regular compensatory behaviors after binging, such as induced vomiting, laxative misuse and prolonged fasting [191]. Therefore, it is frequently associated with overweight or severity of obesity [192]. Commonly, the episode of binge eating occurs on energy dense food that promotes non-homeostatic eating [28], eating in the absence of hunger [193], and the activation of the brain reward systems [194]. Further to the hedonic and addictive value, palatable food becomes a “comfort food”, provoking binge eating behavior, to alleviate anxiety and stress responses [195]. Similarly, to drug abuse, this comfort, especially during dietary periods, encourages to the overconsumption of caloric food [196,197] and sustains the “dark side” of food addiction [198].

### 3.1. The Influence of the DRD4 Gene Polymorphism in Eating Disorders and Obesity

In 1998, Poston et al. was one of the first to investigate an association between the long version of the DRD4 gene and the risk to develop dysfunctional eating patterns, observing that DRD4 L was more frequent in female and male subjects with higher risk for obesity, independently of the degree of body mass index (BMI). They measured the duration of obesity state, parental obesity, severe obesity and novelty-seeking-related scales of the Karolinska Scales of Personality [30]. Although the work was limited only to obese patients with a small range of BMI, without a direct correlation between the Karolinska Scales of Personality and DRD4 long allele, the results of this study revealed an association between this polymorphism and obesity susceptibility.

Subsequently, in two studies [28,29], Levitan et al. investigated a possible role of 7R allele of the DRD4 gene in binge eating behavior and obesity in a group of overeating women with Seasonal Affective Disorder (SAD). SAD is a subtype of Major Depressive Disorder derived from an interaction among biologic disturbances, mood states and seasonal changes [199,200]. Patients with SAD show prevalently hyperphagia and marked craving for high-carbohydrate and high-fat foods, during depressive episodes or negative mood states driving to BED with a consequent weight gain [199,201,202]. Levitan et al. works revealed a significant association between 7R allele and the greater risk of binge eating behavior and overeating that contributes to a high maximal BMI, due to a possible low DA activity in PFC [28,29].

Kaplan et al. continued to study the 7R allele contribution on BMI, analyzing blood samples of women with BN, purging subtype, and they found a positive correlation between this polymorphism and the maximal lifetime BMI [27]. Additionally, it appeared to affect not only the BMI, but even the personality-related psychopathologies common in BN patients, such as increased novelty seeking and impulsivity [190,203].

In accordance with the high prevalence of ED in adolescents and specially in young women [204,205], Sikora study was focused on females. It revealed that women, carriers of at least one copy of DRD4 L allele, reported an increased BMI, and the highest BMI was observed in women 7R/7R homozygotes, in comparison to the other DRD4 polymorphisms [206]. These findings demonstrated that this variant can lead to an excessive food intake to compensate the impaired DA transmission, extending the previously mentioned “reward deficiency syndrome” from drug to food addiction [32,103,206,207]. Other studies highlighted the relationship between the DRD4 gene polymorphism and environmental influences, suggesting that this interaction could play a key role in the promotion of overeating and weight gain.

The season of birth could be considered an environmental factor able to affect eating behaviors; in fact, women with SAD and carriers of the 7R variant, born in spring, exhibited a major risk to develop weight gain and obesity [208], conversely, females with BN and the same allele, showed the highest BMI if born in fall [209]. Additionally, Van Strien et al., studying the influence of the season of birth in males and females with 7R allele of the DRD4 gene, found this effect more pronounced in young women, especially when born in fall, revealing an increased risk of emotional eating [210]. The emotional eating is characterized by a tendency to eat after exposure to stress or negative emotions, preferring sugars and saturated fat items [211], often leading to binge eating [212,213,214].

Furthermore, in two different studies, Silveira et al. showed that preschoolers, carriers of the 7R variant, had different food preferences, depending on sex: girls had less healthy nutrition, eating more fat food than boys [215], suggesting a greater vulnerability to develop weight gain and ED, which could increase if they lived in adverse environments [216]. In support of this last evidence, recent research indicated a reduced risk to maladaptive eating behaviors in children raised in a positive environment with a high predicted prefrontal DRD4 gene expression, measured by an innovative machine learning prediction system (PrediXcan). On the contrary, children with the same predicted gene expression, living in a less positive environment, developed obesogenic behaviors, including overconsumption of high sugar drinks and emotional eating [217]. Moreover, another relationship between a negative environmental factor and the variation of the DRD4 gene is represented by a history of low maternal sensitivity in girls with the 7R allele, promoting overweight and thus the risk to become obese [218].

Taken together, this evidence shows that the 7R allele seems to increase the susceptibility to multiple environmental factors, and thus, individuals, carriers of this polymorphic variant, are more prone to overweight and dysfunctional eating patterns. Furthermore, the polymorphism of the DRD4 gene is associated with reward seeking behaviors: adults with versus without the 7R variant exhibited a greater craving in response to food cues, predisposing them to binge eating behavior [146]. Then, Stice et al. observed, through an fMRI study, a correlation between the 7R allele and reduced activity in the reward circuitry in adolescent girls, with a different range of BMI, in response to palatable food imagines, predicting unhealthy weight gain [219]. Comparing overweight with adolescent lean girls, the first ones exhibited more activity in the ventrolateral PFC, a factor which is positively correlated with BMI, due to the role of this area on directing attention to palatable food [219,220]. In a following study, always using the fMRI paradigm, Stice et al. continued to investigate the brain response to receipt and anticipated receipt of palatable food in lean adolescents, with different genotypes related to low dopaminergic transmission, including 7R or longer allele. Alterations in cerebral areas sensitive to food reward were found in these adolescents, which might perceive the food reward more salient [221].

The polymorphism of the DRD4 gene not only influences BMI and overeating but also the development of AN and correlated psychopathological features. Supporting this hypothesis, several researches found a strong association between the 7R genotype and the risk of AN [222]. Probably, this association is caused by disturbances in the food reward circuitries [223] and by the involvement of DRD4 gene polymorphism in the psychopathological features, especially perfectionism and body dissatisfaction in patients with AN [26]. The various studies that investigated the effect of the DRD4 polymorphism are listed in Table 2.

Despite these positive correlations, it is important also to consider contrasting results on the effect of DRD4 gene polymorphism in AN and the other ED.

For instance, Hinney et al. found that the polymorphism of the DRD4 gene was not statistically relevant to the etiology of AN and obesity in German adolescents, but the study had the limit of not considering healthy subjects as a control group [224]. Guo et al. showed a reduction in BMI in African American and Hispanic young adolescent siblings, carriers of the 7R/7R genotype [225,226]. In addition, Fontana et al. found a higher BMI, energy intake and waist circumference in preschooler children without 7R allele compared to 7R carriers [227]. Accordingly, other studies corroborated no implication of DRD4 L on increased BMI [228,229]. Karwautz et al. described a non-significant association between DRD4 gene variants and AN, comparing 45 sister-pairs, one with AN and the other without any ED [230]. Furthermore, Yilmaz et al. observed a correlation between the 7R allele of the DRD4 gene and BN only if these patients had a history of childhood ADHD [231]. Finally, considering that the peripheral expression of dopaminergic genes reflects the brain status, Frieling et al. analyzed blood of patients suffering from ED and did not find a significant relationship between DRD4 gene and AN or BN [232]. These conflicting studies and the respective outcomes were shown in Table 3.

### 3.2. Preclinical Studies

Taking into account these controversial results and the possible influence of DRD4 gene in eating behaviors and in the regulation of body weight in human studies, it would be interesting to deeply explore the role of the DRD4 in well characterized animal models of aberrant eating patterns.

Until now, very few preclinical studies have been done. Compared to WT, mutant mice lacking DRD4 showed similar behavioral responses in standard food pellets consumption under self-administration paradigm [233], and they did not reveal significant differences on the reinforcing effectiveness of food [234]. Other researches evidenced that clozapine, an atypical antipsychotic and a DRD4 antagonist, increased food intake in rats [235] and mice [236]. These results are consistent with clinical studies in which clozapine stimulates appetite and craving for carbohydrates and palatable food, leading to episodes of binge eating [237,238] and weight gain because of the inability to suppress hyperphagia [239]. However, clozapine is not a selective DRD4 antagonist but has affinity for receptors of multiple neurotransmitters including serotonin, noradrenaline and histamine [240], and this could be the reason for the effect on appetite [241].

Afterwards, Huang et al. evaluated the mRNA DRD4 expression in the subcortical areas of the brain, in obesity prone or resistant mice, under chronic high-fat diet [242]. Using in situ hybridization techniques, compared to control (mice fed with low-fat diet) and obesity resistant mice, obesity prone mice had significantly higher levels of DRD4 mRNA expression in the ventral part of the lateral septal nucleus and ventromedial hypothalamic nucleus, suggesting a role for this receptor in the hypothalamic pathway [242]. Indeed, the DRD4, despite being predominantly expressed in the PFC, is also importantly localized in the hypothalamus [37,242], and it is well known that hypothalamic circuitries contribute to appetite control and energy homeostasis [243,244,245,246,247] by integrating a variety of neural inputs arising from both neuropeptides and neurotransmitters [248,249,250,251,252,253,254,255] and modulating the mechanisms underlying ingestive behavior and stress [256,257,258]. These findings suggest that the DRD4 may influence food intake and eating behaviors at the level of both the PFC, as previously discussed, and the hypothalamus. Interestingly, stimulation of the DRD4 with the selective agonist PD-168,077, directly injected in the paraventricular nucleus of the hypothalamus (PVN), induced hyperphagia in male rats, apparently caused by presynaptic inhibition of glutamate release in the PVN and, at the same time, it decreased plasma corticosterone levels in food-restricted rats [259]. Glutamate directly stimulates CRF neurons [260]. Thus, the authors speculated that the hyperphagia was induced by synergistic interaction among DRD4, glutamate and CRF.

Intriguingly, this effect was completely reversed by the pretreatment with the DRD4 antagonist L-745,870 that was inactive per se on food intake [260]. In another study [129], the same DRD4 antagonist had no effect on reinstatement of food seeking behavior in standard food pellets, elicited by food cues or food priming.

## 4. Conclusions

The findings herein support a potential relationship among DRD4 and the gene polymorphism, drug and food addiction. The localization and connectivity of DRD4, predominant in PFC and hypothalamus and related neuronal pathways present in these brain regions, in addition to DRD4 involvement on personality traits, especially novelty seeking and impulsivity, suggest a complex action of this DA receptor in addicted behavior. Consequently, the deficiency in DRD4 signaling, particularly evident in the 7R variant of the gene, could increase the susceptibility to the development of substance abuse or ED and related comorbidities. The DRD4 was intensively investigated in human studies and considerably less in animals, mostly in mutant mice lacking DRD4 and in the context of drug addiction. Genetic association studies are important to understand the role played by the DRD4 gene in the disorders discussed in this review; however, further research will be necessary to explain the controversial results debated above, especially a deep investigation using preclinical studies. It is important to consider, for instance, the compensatory changes observed in the brain of DRD4 KO mice and to extend the research to validated animal models of ED, in order to evaluate the effect of selective DRD4 agonists and antagonists on feeding behavior. To the best of our knowledge, the effect of DRD4 compounds on palatable food, as well as the possible role of DRD4 and adenosine A_2A_ receptor (A_2A_AR) heteromers [261,262,263] on compulsive behavior, has been unexplored since the promising results of A_2A_AR agonists to block binge eating behavior and drug abuse [264,265,266,267,268]. Similarly, it would be interesting to investigate the interaction between sigma and DRD4 pathway, taking into account that they both modulate glutamate release and several DRD4 compounds bind also sigma1 receptors with high affinity [122,269,270]. Moreover, recent works highlighted the strong effect of sigma1 receptor antagonists to attenuate many behaviors related to drug abuse and binge eating episodes [271,272,273,274].

In summary, this review highlights the attractive role of DRD4 as an interesting pharmacological target and promotes the discovery of innovative selective compounds for the treatment of addictive disorders and their related comorbidities.

## Figures and Tables

**Table 1:** Dopamine D4 receptor (DRD4) compounds in drug addiction.

Compounds	Type of Interaction	Species	Effect	References
L-745,870	Antagonist	Rats	Reduction of methamphetamine-induced discriminative effect	[125]
		Mice	Reduction of morphine-induced withdrawal	[127]
		Rats	Reduction of reinstatement of both nicotine- and cue-induced nicotine seeking behavior	[129]
		Rats	Reduction of alcohol self-administration and stress-induced reinstatement	[128]
L-750,667	Antagonist	Rats	Does not attenuate reinstatement of cocaine-seeking behaviors	[133]
PNU-101387G	Antagonist	Rats	Prevention of amphetamine induced-behavioral sensitization	[134]
ABT-724	Partial Agonist	Rhesus Monkeys	Does not maintain the rates of cocaine self-administration behavior	[144]
PD-168,077	Agonist	Rats	Decrease of morphine-induced hyperlocomotion, reward and withdrawal syndrome	[137]
A-412997	Agonist	Rats	Does not induce CPP compared to amphetamine and methamphetamine	[145]

CPP: conditioned place preference.

**Table 2:** The effect of the 7R or longer allele (DRD4 L) in eating patterns.

Subjects of Study	Genotype	Result	Reference
Patients with BMI >30	DRD4 L vs. DRD4 S	Dysfunctional eating patterns that promote obesity	[30]
SAD women	7R vs. no 7R allele	Binge eating behavior and increased BMI	[28,29]
BN women	7R vs. no 7R allele	Increased BMI	[27]
BN patients	7R vs. no 7R allele	Affect personality-related psychopathologies	[203]
BN patients	7R vs. no 7R allele	Affect personality-related psychopathologies	[190]
Women	DRD4 L vs. DRD4 S	Increased BMI	[206]
SAD women	7R vs. no 7R allele	Weight gain and obesity in SAD women born in spring	[208]
BN women	7R vs. no 7R allele	High BMI in BN women born in fall	[209]
Young women	7R vs. no 7R allele	Emotional eating in young women born in fall	[210]
4 years old children	7R vs. no 7R allele	Susceptibility to develop weight gain and ED	[215]
Preschooler children	7R vs. no 7R allele	Risk of obesity in women with history of low maternal sensitivity	[218]
Individuals with and without symptoms of binge eating	DRD4 L vs. DRD4 S	Craving predisposing to binge eating behavior	[146]
Lean to obese adolescent female	7R vs. no 7R allele	Unhealthy weight gain	[219]
AN women	7R vs. no 7R allele	Risk to develop AN	[222]
AN women	7R vs. no 7R allele	Affect personality-related psychopathologies	[26]

AN: Anorexia Nervosa; BMI: Body Mass Index; BN: Bulimia Nervosa; DRD4 L: Dopamine D4 receptor long allele; DRD4 S: Dopamine D4 receptor short allele; ED: Eating Disorder; SAD: Seasonal Affective Disorder; 7R: 7-repeat.

**Table 3:** The effect of the 7R or longer allele (DRD4 L) in eating patterns.

Subjects of Study	Genotype	Result	Reference
Adolescent and young adult patients with acute AN; underweight students; children and adolescents with obesity	Allelic variants of DRD4 gene	No evidence in etiology of AN and obesity	[224]
Adolescents and young adults	7R vs. no 7R allele	Reduction in BMI in African American and Hispanic individuals	[225,226]
Children	7R vs. no 7R allele	Lower BMI, energy intake and waist circumference	[227]
Overweight and obese compared to normal weight adolescents	7R vs. no 7R allele	No evidence in increased BMI and risk of obesity	[228]
Adolescents from lean to obese	DRD4 L vs. DRD4 S	No evidence in increased BMI and risk of obesity	[229]
Sister pairs: one with AN and the other one with no history of any form of ED	Allelic variants of DRD4 gene	No correlation with AN	[230]
Women with current or past BN purging subtype, and a subgroup of them with a childhood ADHD	7R vs. no 7R allele	No correlation with BN, if not associated with ADHD	[231]

AN: Anorexia Nervosa; ADHD: Attention deficit hyperactivity disorder; BMI: Body Mass Index; BN: Bulimia Nervosa; DRD4: Dopamine D4 receptor; DRD4 L: Dopamine D4 receptor long allele; DRD4 S: Dopamine D4 receptor short allele; ED: Eating Disorder; 7R: 7-repeat.
